# Open Globe Injury Secondary to “Durian” Fruit Fall

**DOI:** 10.7759/cureus.19978

**Published:** 2021-11-29

**Authors:** Ainul Basirah Ibramsah, Nurul Ain Masnon, Mohtar Ibrahim, Wan-Hazabbah Wan Hitam

**Affiliations:** 1 Department of Ophthalmology and Visual Sciences, Universiti Sains Malaysia School of Medical Sciences, Kota Bharu, MYS

**Keywords:** penetrating ocular injuries, open globe injury, ocular emergency, protective device, durian

## Abstract

A devastating ocular injury can be caused by durian, the “king of fruits.” We report a case of open globe injury secondary to a durian fruit fall. A 54-year-old unfortunate male was accidentally hit by a durian fruit that fell from a tree. The fruit hit directly his right face and eye. He experienced a transient loss of consciousness. He sustained extensive corneal and multiple scleral lacerations with total hyphema, iridodialysis, posterior dislocation of the lens, and retinal detachment. There were also multiple sites of a puncture wound on the right side of the face and right upper lid laceration. Primary suturing of corneal and scleral laceration with anterior chamber washout was performed. The right visual acuity remained poor postoperatively. Durian fruit injury to the eye may lead to severe devastating ocular complications that lead to blindness. The prognosis depends on the severity of the injury.

## Introduction

Durian or *Durio zibethinus* is a seasonal tropical thorny fruit and is known as the “king of fruits” in Southeast Asia. “Duri” means thorn in Malaysia. It is also known as duren (Indonesian), duyin (Burmese), thureen (Cambodian), thurian (Thai), saurieng (Vietnamese), dulian (Philippines), stinkvrucht (Dutch), and kadu (Sudan). It has a unique flavor but poses a strong onion-garlic aroma, which is offensive to some. It contains high nutrients with bioactive compounds such as anthocyanins, flavonoids, carotenoids, and flavanols and great antioxidant and anti-inflammatory properties [1]. Durian trees are tall and can reach up to 40 m in height. The shape of durian is either oblong or round, and the rind is thick, tough, semiwoody, and densely set with stout, sharply pointed spines. The size can reach 30 cm in length and 15 cm in diameter, and the weight can reach up to 8 kg [2]. Because of the tree height and the sharp thorns and weight of this fruit, it can cause high-velocity penetrating or blunt ocular injury to people passing by under the tree. In contrast to other thorny fruit harvesting industries such as prickly pears and date palms, the reported ocular injuries caused by them were less severe and did not extend beyond the lens. We report a devastating globe rupture caused by a durian fruit fall. So far, there were only four cases of ocular durian injury reported [3,4].

## Case presentation

A 54-year-old male was brought to the emergency department for a sudden loss of vision in the right eye after being hit by a durian fruit. The incident occurred while he was walking under a durian tree at the back of his house. All of a sudden, a big size durian fruit (about 3 kg) fell from a tree that is about 12 feet tall. The fruit hit his right face and eye. He experienced a transient loss of consciousness for about five minutes. Subsequently, he noticed that his right vision was poor. He was not wearing any head or eye protection. He was brought immediately by his family to the hospital.

**Figure 1:**
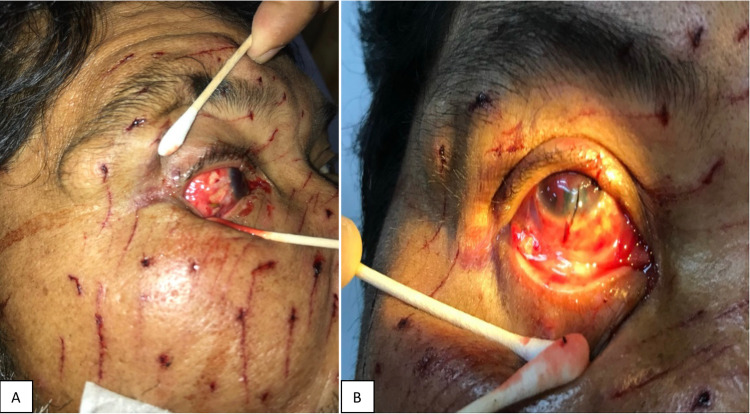
Multiple superficial puncture wounds over the right side of the face (A) and right upper lid laceration with corneal and scleral laceration wound and traumatic hyphema (B).

On examination, there were multiple puncture abrasions over the right cheek and forehead. He also sustained a right upper lid laceration. The visual acuity in the right eye was light perception (LP) with a positive afferent pupillary defect, while the left visual acuity was 6/6. There were multiple corneoscleral lacerations with total hyphema (Figure 1). No iris prolapse or iris details was seen. There was no fundal view in the right eye. The right eyeball was soft. The left anterior segment was normal with a normal fundus appearance. The patient was diagnosed to have an open globe injury in the right eye.

**Figure 2:**
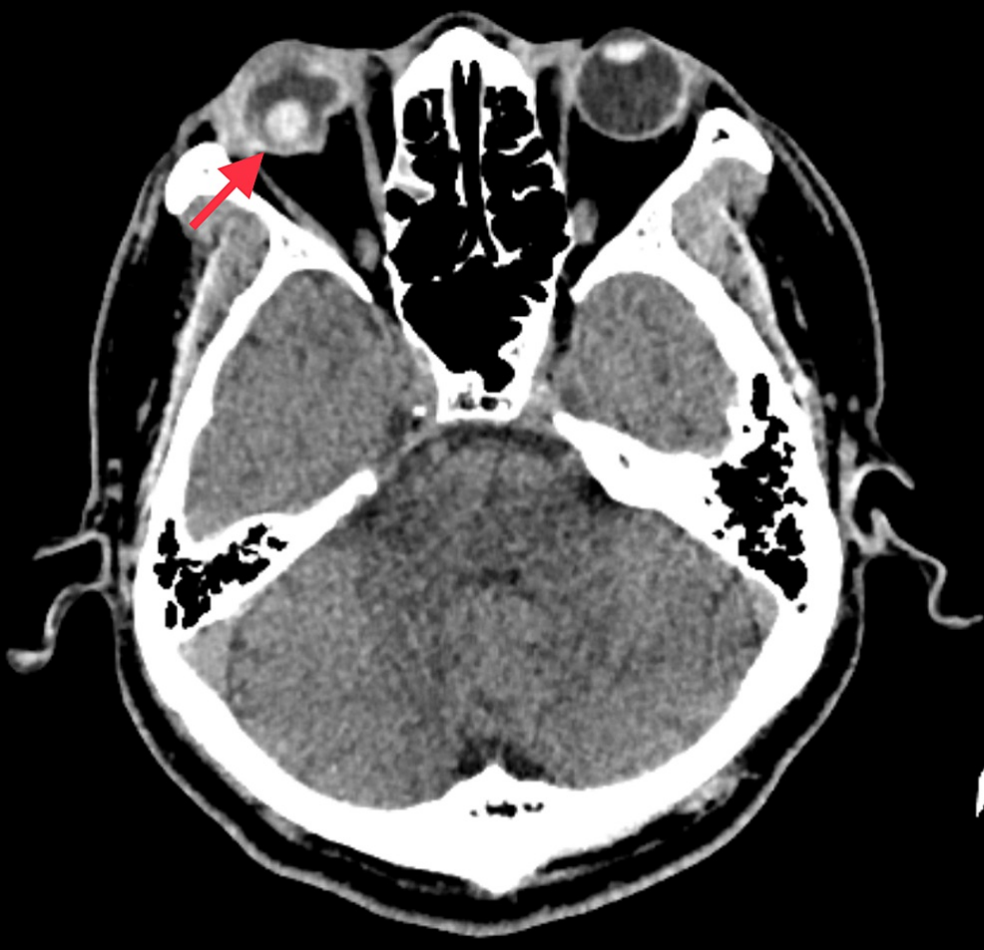
Axial cut of CT scan shows right globe rupture with posteriorly dislocated crystalline lens (red arrow).

Urgent computed tomography (CT) scan demonstrated a “flat tire sign,” indicating globe rupture with posteriorly dislocated lens (Figure 2). The skull and orbital wall were intact with no fracture seen. There was no intracranial bleed.

**Figure 3:**
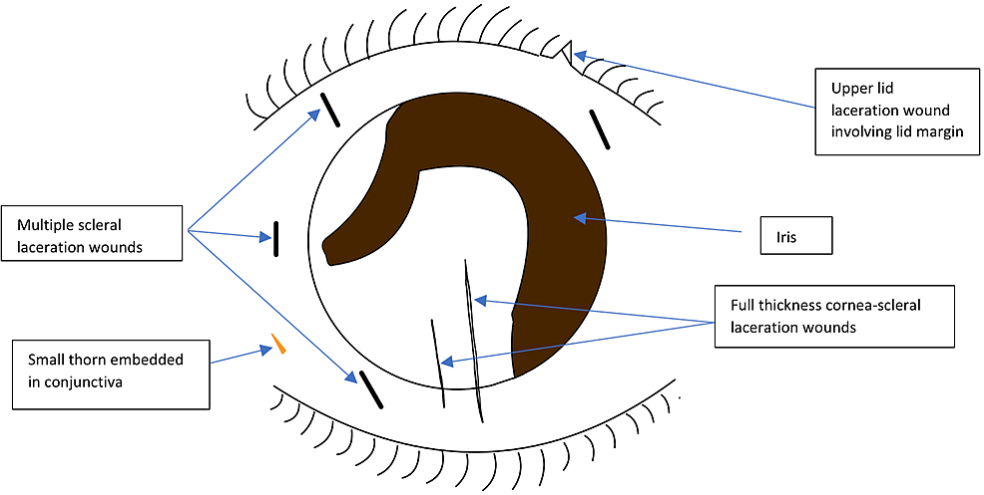
Illustration of intraoperative findings showing the sites of the corneal and scleral lacerations with loss of the half of the iris tissue inferiorly and iridodialysis at 9–11 o’clock.

**Figure 4:**
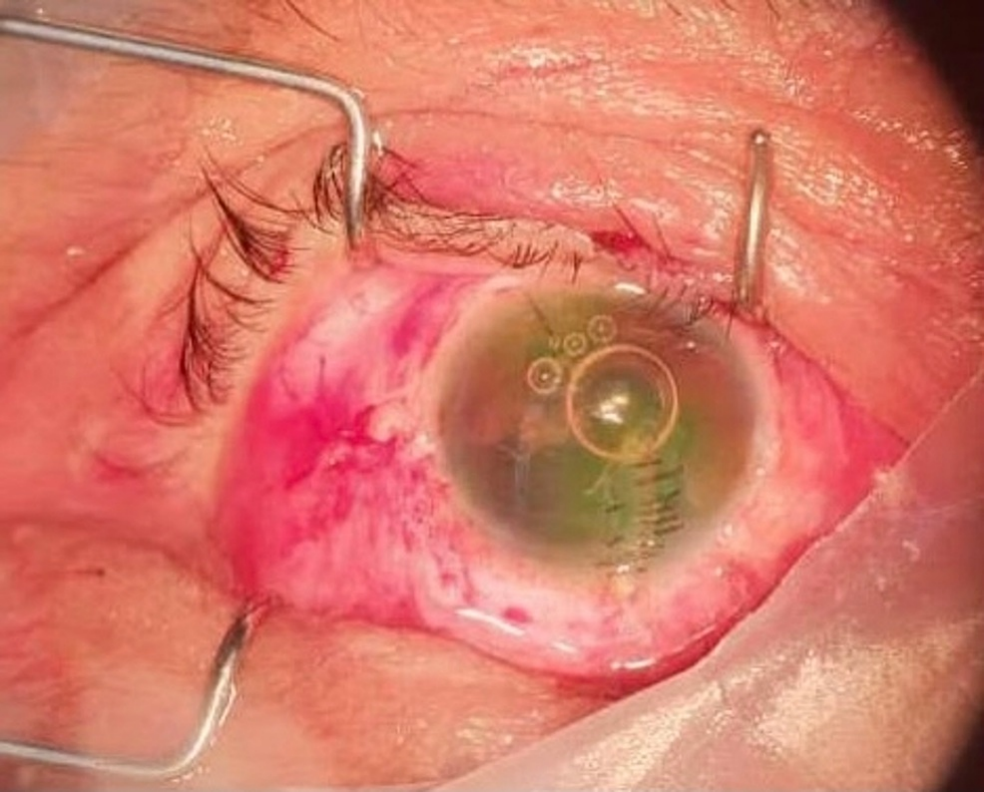
Post corneal-scleral laceration wound repair and anterior chamber washout.

The patient was planned for an emergency operation under general anesthesia. A 360-degree conjunctival exploration was performed. There was a small piece of thorn measuring 3 mm embedded in the conjunctiva that was removed. The corneal and scleral wounds were sutured. Anterior chamber washout was performed. The details of the ocular injuries are shown in the diagram in Figure 3. Upper lid laceration was sutured in layers. Image post repair is shown in Figure 4. Intravitreal vancomycin and ceftazidime were administered. On further exploration, there was no visible scleral laceration up to the equator of the eyeball.

The patient was started on systemic ciprofloxacin for a total duration of two weeks along with topical moxifloxacin and prednisolone acetate. There were no signs of infections that developed postoperatively with the medications. Postoperatively, his visual acuity remained LP. The patient developed recurrent episodes of hyphema. The eyeball was firm with intraocular pressure of 16 mmHg. Ultrasound B-scan revealed a total retinal detachment with extensive vitreous hemorrhage. The prognosis of the affected eye was discussed with the patient. He was also warned regarding complications of sympathetic ophthalmia. However, he was not keen on further intervention.

On follow-up at one month, his visual acuity remained LP. The right anterior chamber was quiet with the presence of iridodialysis. There was no hyphema, and the intraocular pressure was 11 mmHg. The visual acuity in the right eye was persistently poor after six months of follow-up. Ultrasound B-scan showed fibrovascular traction that developed over the retinal detachment. There was no vitreous hemorrhage.

## Discussion

Based on the review of ocular injuries, the common mode of ocular injury was due to sharp objects, motor vehicle accidents, firecrackers, and chemicals. Few rare ocular injuries were reported to be caused by superglue and durian fruit [3]. From our literature review, there were only four cases of ocular durian injury reported that involved patients from the age range of 12-40 years old (Table 1) [4,5]. Our patient is older than the age range reported (54 years old).

**Table 1:** Summary of previous literature on ocular injuries due to durian fruit. Abbreviations: VA, visual acuity; LP, light perception; HM, hand movement; CF, counting fingers; VH, vitreous hemorrhage; AC, anterior chamber; FB, foreign body; EUA, examination under anesthesia; RD, retinal detachment

No.	Author, year	Age	Gender	Activity, time of injury	Laterality of facial or ocular injury	Initial VA	Ocular findings	Interventions	Final VA
1	Aziz et al., 2009 [4]	40	F	Collecting ripe durian fruits, NA	Right	LP	Lid laceration, corneal laceration, iris prolapse, lens extrusion, hyphema, and VH	Lid suturing, corneal suturing, and AC washout	LP
2	Reddy, 2012 [5]	40	M	Resting under a tree, 4:45 pm	Right	HM	Corneal laceration, iris incarceration, total hyphema, and scleral FB	Corneal suturing, AC washout, and removal of scleral FB	6/18
		20	M	Sleeping under a tree, night	Left	3/60	Corneal laceration with iris prolapse	Corneal suturing and prolapsed iris reposition	6/6
		12	M	Playing with a friend under a tree, 5:30 pm	Left	CF	Scleral FB, traumatic mydriasis, corneal abrasion, and commotio retinae with macula edema	EUA and removal of scleral FB	6/6
3	Present case, 2021	54	M	Walking under a tree, 2:00 pm	Right	LP	Lid laceration, subconjunctival FB, corneal and scleral laceration, hyphema, lens dislocation, VH, and RD	Lid suturing, corneal and scleral suturing, and AC washout	LP

In contrast to other thorny fruits, ocular durian injuries are known to be more severe. Studies of ocular injuries due to date palm thorns and prickly pear spine showed mostly these thorny fruits causing keratitis, corneal ulcer, and corneal perforation with no injury beyond the lens. Both studies also show that eye injuries occur mainly due to a lack of protective eyewear [6,7]. Our patient demonstrated severe ocular injury caused by durian. The patient sustained multiple scleral and corneal lacerations, puncture lacerations, hyphema, iridodialysis, dislocation of the lens, and retinal detachment.

CT scan is an important tool in ocular trauma in detecting orbital wall fracture, globe rupture, and intraocular foreign body. CT scan helped show the posteriorly dislocated crystalline lens and globe rupture where the fundus was poor. However, ultrasound is regarded as more superior in detecting the severity of globe injuries, such as the presence of retinal detachment, vitreous hemorrhage, and endophthalmitis, which are not directly visible with computed tomography [8]. Ultrasound B-scan improved visualization of orbital structures obscured by opaque media. Since the machine is not portable in our center, the role of ultrasound B-scan in this patient was limited during the initial presentation. However, postoperatively, it helped diagnose the presence of retinal detachment and monitor the progression of vitreous hemorrhage.

Previous reports have shown that most durian ocular injuries required surgical interventions [3]. Without evidence of penetrating injury under the slit lamp, a meticulous examination under general anesthesia may still be warranted, especially in pediatrics or noncooperative patients. This is to rule out penetrating scleral injury or retained piece of durian thorn that may predispose to ocular infections such as conjunctiva or scleral abscess, as well as endophthalmitis. We were able to explore up to the equator to look for any scleral perforating injury in this patient.

Antibiotics have been routinely administered in open globe injuries as a prophylaxis to prevent post-traumatic endophthalmitis. It can be given systemically, intravitreally, or topically. A randomized control trial in 2017 conducted in open globe injury patients who received prophylaxis intravenous and oral antibiotics compared with those who received oral antibiotics shows no statistically significant difference between the two arms; the rates of post-traumatic endophthalmitis were 2.1% and 2.2%, respectively [9]. Our patient received intravitreal and oral and topical antibiotics and showed no signs of endophthalmitis up to six months of follow-up.

Visual outcome was poor in our patient due to the extensive nature of the ocular injury. This is consistent with previous findings that open globe injury, poor initial visual acuity, the length and severity of the corneal and scleral wound, the presence of hyphema, lens injury, and retinal detachment are predictive of poorer visual outcomes in ocular trauma [10]. Patients with open globe injury need longer follow-up as they have a high risk to develop sympathetic ophthalmia. It is a devastating complication in ocular trauma as it can cause blindness to the normal eye. We monitored our patient for up to six months, and so far, there is no sign of sympathetic ophthalmia.

## Conclusions

Durian fruit injury to the eye may lead to severe devastating ocular complications that may lead to blindness. The prognosis depends on the severity of the injury. A proper ocular protective device is encouraged to be used by those involved in handling durian farms and industry to prevent unfortunate events, especially ocular injuries. We hope that this case will be an eye-opener to the community, especially those who are involved in the farming industry, to always practice personal protective measures.
